# Adaptative Skills and Global Functioning of Unaccompanied Migrant Minors in Europe: A Systematised Review

**DOI:** 10.3389/ijph.2024.1606625

**Published:** 2024-06-26

**Authors:** Dimitri Prod’hom, Joëlle Rosselet Amoussou, Kerstin Jessica Plessen, Noémie Cuissart de Grelle, Sydney Gaultier

**Affiliations:** ^1^ Lausanne University Hospital and University of Lausanne, Lausanne, Switzerland; ^2^ Faculty of Biology and Medicine, University of Lausanne, Lausanne, Switzerland; ^3^ Division of Child and Adolescent Psychiatry, Lausanne University Hospital and University of Lausanne, Lausanne, Switzerland; ^4^ Child and Adolescent Psychiatry Clinical Services, Geneva University Hospital, Geneva, Switzerland

**Keywords:** unaccompanied minors, education, adaptation, mental health, resilience

## Abstract

**Objectives:**

This systematised review aimed to examine European literature reporting data about adaptative skills and global external functioning of unaccompanied minors (UAMs).

**Methods:**

We conducted a systematised screening of four databases (APA PsycINFO Ovid, Medline Ovid ALL, Embase.com and Web Of Science Core Collection) using a research strategy including social, scholarly and behavioural abilities as well as externalising problems associated with the target population of UAMs. Thirty articles were included using pre-defined inclusion and exclusion criteria.

**Results:**

Our review showed that despite high levels of internalising disorders, socio-behavioural and educational adjustment of UAMs remained positive. It demonstrated how this population displays a strong desire for academic success and prosocial behaviours instead of aggressivity in everyday life. Nevertheless, our review drew attention to the strong tendency of UAMs to internalise their disorders and display chronic distress and problematic behaviours which increased with time spent in the host country.

**Conclusion:**

Our study draws attention to the risk of underestimating the real mental health needs of refugees, due to preserved external functioning combined with significant settlement pressures.

## Introduction

In the context of migration, the United Nations Committee on the rights of the child defines unaccompanied minors as children “who have been separated from both parents and other relatives and are not being cared for by an adult who, by law or custom, is responsible for doing so.” Concerning mental health, the prevalence of depression, anxiety and posttraumatic stress disorder (PTSD) is high among UAMs and persists over time. Although the population of UAMs is very heterogeneous between the studies, the higher mental health risk than in adult and accompanied minor (AM) refugees is well established [1]. The prevalence of PTSD among UAMs in Europe has been evaluated at between 19% and 52.7%, with a median of 35.5% [2]. A Swedish study comparing 710,170 native minors to 33,501 UAMs showed an 8-fold higher risk of PTSD in the second group [3].

UAMs are more exposed to traumatic life events because of their younger age and lack of parental protection. They also face high levels of psychosocial and administrative insecurity, particularly during the transition to adulthood [4]. Psychosocial stress factors contribute to the persistence and worsening of their disorders [5]. Even with the support of healthcare professionals, UAMs still face important social pressures regarding their independence and transition to adulthood which can hinder their adaptation and global functioning in a new country.

Adaptative skills are defined by the American Psychology Association (APA) as “abilities that enable one to meet new challenges, such as the ability to adjust to a new environment and to learn new things.” They also involve “self-management, such as the ability to control one’s impulses.” [6] Hence, in children, adaptative skills may be represented by the presence or absence of externalised disorders, academic and educational achievement as well as interpersonal skills. We therefore used these concepts as a framework for our search strategy.

While studies of externalising disorders (irritability, aggressive behaviours, poor peer relations, etc.) in UAMs report heterogenous prevalence rates [7–11], many studies describe a tendency in youth with immigrant backgrounds [12–14], particularly UAMs, to internalise their emotional problems and mental health needs. These results suggest the possible existence of a specific form of “external resilience” among young refugees and migrants [15–18].

Secondly, evidence suggests that academic achievement may be negatively linked with the presence of PTSD or depression in the general population [19–21]. However, this is not always confirmed in studies of refugee populations. Some studies report a higher risk of school problems among refugees [22, 23], while others observe a particular form of resilience to these problems [24–26]. Furthermore, the adaptative skills examined in these studies usually include other spheres of social and professional life [14, 24], suggesting a high level of general functioning in this population.

Explaining these findings requires taking into account social issues relating to the hosting and integration of refugees, as well as screening and access to mental healthcare. For refugees facing administrative insecurity, the negative consequences of externalised distress are significant. Conversely, access to care and screening of emotional distress are hampered when troubles are internalised.

This systematised review aims thus to clarify the adaptative skills of UAMs in European host countries and particularly social, academic and learning capacities as well as prevalence of externalising disorders and global functioning difficulties.

## Methods

### Search Strategy

A literature search of four bibliographic databases, Embase.com, Ovid MEDLINE(R) ALL, APA PsycINFO Ovid and Web of Science Core Collection, was conducted in September 2022. All searches were carried through without restrictions of language or date. Additional records were identified through backward citation tracking and Google Scholar. The detailed search strategies, keywords, and index terms are presented in Table 1.

**TABLE 1:** Bibliographic database search strategies (Adaptative skills and global functioning of unaccompanied migrant minors in Europe: a systematised review, Lausanne, Switzerland, 2022–2024).

Embase.com
197 results, September 09, 2022 (unaccompanied AND (minor* OR child* OR adolescent* OR youth* OR young OR teen*)):ab,ti, kw AND ('social behavior'/de OR 'social adaptation'/de OR 'maladjustment'/de OR 'social communication'/de OR 'social inclusion'/de OR 'social exclusion'/exp OR 'social competence'/de OR 'social interaction'/de OR 'social discrimination'/de OR 'social cognition'/de OR 'social disability'/de OR 'integration'/de OR 'adjustment'/de OR 'academic achievement'/exp OR 'learning disorder'/de OR 'educational attainment'/de OR 'educational status'/de OR 'high school'/exp OR 'social health'/de OR 'human relation'/de OR 'adaptability'/de OR 'flexibility'/de OR 'culture change'/de OR 'cross cultural adaptation'/de OR 'interpersonal communication'/de OR 'adaptive behavior'/de OR 'psychological adjustment'/exp OR 'adjustment disorder'/de OR 'behavior disorder'/de OR 'conduct disorder'/exp OR 'externalizing disorder'/exp OR 'antisocial behavior'/exp OR ("peer relation*" OR socio-behav* OR sociobehav* OR (social NEXT/1 (behav* OR action OR activity OR activities OR contact* OR communication OR interaction* OR adapt* OR functioning OR responsiveness OR sensitivity OR health OR cognition OR exchange OR inclusion OR integration OR relation* OR competence* OR skill* OR abilit* OR disabilit* OR breakdown OR dysfunction OR handicap OR discrimination OR issues OR processes)) OR "adaptive behav*" OR (behav* NEXT/3 (disorder* OR disturbance* OR problem*)) OR externali* OR "conduct disorder*" OR ((anti-social OR antisocial) NEXT/1 behav*) OR "socioemotional functioning" OR "human relation*" OR marginali?ation OR (interpersonal NEXT/1 (skill* OR competence* OR interaction* OR relation* OR communication)) OR adaptability OR flexibility OR maladjustment OR "social maladapt*" OR adjustment OR adaptation OR acculturation OR "cultural assimilation" OR "culture change" OR "sociocultural factor*" OR ("cross cultural" NEXT/1 (adapt* OR communication)) OR ((intercultural OR interethnic) NEXT/1 communication) OR "adaptive behav*" OR (academic NEXT/1 (achievement* OR underachievement OR success OR performance* OR failure*)) OR (school NEXT/1 (belonging OR achievement OR success OR behav* OR performance* OR graduation OR learning OR engagement OR integration)) OR "high school" OR (educational NEXT/1 (resilience OR attainment OR standard* OR status)) OR integration OR (learning NEXT/1 (disorder* OR deficit OR difficult* OR disabilit* OR impairment OR problem* OR disturbance*)) OR "impaired learning"):ab,ti,kw) NOT [conference abstract]/lim

Medline Ovid ALL
Ovid MEDLINE(R) ALL 1946 to September 09, 2022 141 results (unaccompanied AND (minor* OR child* OR adolescent* OR youth* OR young OR teen*)).ab,ti,kf. AND (Social Behavior/OR Social Adjustment/OR social inclusion/OR Social Isolation/OR social skills/OR Social Interaction/OR Social Discrimination/OR Social Cognition/OR exp Social Integration/OR exp Academic Performance/OR Learning Disabilities/OR Educational Status/OR Communication/OR Adjustment Disorders/OR exp Adolescent Behavior/OR exp child behavior/OR Child Behavior Disorders/OR Problem Behavior/OR Conduct Disorder/OR exp Emotional Adjustment/OR Acculturation/OR Social Marginalization/OR ("peer relation*" OR socio-behav* OR sociobehav* OR (social ADJ (behav* OR action OR activity OR activities OR contact* OR communication OR interaction* OR adapt* OR functioning OR responsiveness OR sensitivity OR health OR cognition OR exchange OR inclusion OR integration OR relation* OR competence* OR skill* OR abilit* OR disabilit* OR breakdown OR dysfunction OR handicap OR discrimination OR issues OR processes)) OR "adaptive behav*" OR (behav* ADJ3 (disorder* OR disturbance* OR problem*)) OR externali* OR "conduct disorder*" OR ((anti-social OR antisocial) ADJ behav*) OR "socioemotional functioning" OR "human relation*" OR marginali#ation OR (interpersonal ADJ (skill* OR competence* OR interaction* OR relation* OR communication)) OR adaptability OR flexibility OR maladjustment OR "social maladapt*" OR adjustment OR adaptation OR acculturation OR "cultural assimilation" OR "culture change" OR "sociocultural factor*" OR ("cross cultural" ADJ (adapt* OR communication)) OR ((intercultural OR interethnic) ADJ communication) OR "adaptive behav*" OR (academic ADJ (achievement* OR underachievement OR success OR performance* OR failure*)) OR (school ADJ (belonging OR achievement OR success OR behav* OR performance* OR graduation OR learning OR engagement OR integration)) OR "high school" OR (educational ADJ (resilience OR attainment OR standard* OR status)) OR integration OR (learning ADJ (disorder* OR deficit OR difficult* OR disabilit* OR impairment OR problem* OR disturbance*)) OR "impaired learning").ab,ti,kf.)

APA PsycINFO Ovid
APA PsycInfo 1806 to September Week 1 2022 394 results, September 09, 2022 (unaccompanied AND (minor* OR child* OR adolescent* OR youth* OR young OR teen*)).mp. AND (social behavior/OR social functioning/OR social adjustment/OR social communication/OR social inclusion/OR social skills/OR exp social interaction/OR social discrimination/OR exp social cognition/OR exp social integration/OR exp adjustment/OR exp academic achievement/OR academic aptitude/OR school graduation/OR school learning/OR exp learning disorders/OR educational attainment level/OR educational standards/OR high schools/OR high school education/OR social health/OR interpersonal relationships/OR "adaptability (personality)"/OR exp culture change/OR cross cultural communication/OR interpersonal communication/OR adaptive behavior/OR adjustment disorders/OR "emotional and behavioral disorders"/OR behavior disorders/OR conduct disorder/OR behavior problems/OR externalizing symptoms/OR exp antisocial behavior/OR socioemotional functioning/OR social issues/OR social processes/OR marginalization/OR interpersonal interaction/OR social exchange/OR exp culture change/OR sociocultural factors/OR ("peer relation*" OR socio-behav* OR sociobehav* OR (social ADJ (behav* OR action OR activity OR activities OR contact* OR communication OR interaction* OR adapt* OR functioning OR responsiveness OR sensitivity OR health OR cognition OR exchange OR inclusion OR integration OR relation* OR competence* OR skill* OR abilit* OR disabilit* OR breakdown OR dysfunction OR handicap OR discrimination OR issues OR processes)) OR "adaptive behav*" OR (behav* ADJ3 (disorder* OR disturbance* OR problem*)) OR externali* OR "conduct disorder*" OR ((anti-social OR antisocial) ADJ behav*) OR "socioemotional functioning" OR "human relation*" OR marginali#ation OR (interpersonal ADJ (skill* OR competence* OR interaction* OR relation* OR communication)) OR adaptability OR flexibility OR maladjustment OR "social maladapt*" OR adjustment OR adaptation OR acculturation OR "cultural assimilation" OR "culture change" OR "sociocultural factor*" OR ("cross cultural" ADJ (adapt* OR communication)) OR ((intercultural OR interethnic) ADJ communication) OR "adaptive behav*" OR (academic ADJ (achievement* OR underachievement OR success OR performance* OR failure*)) OR (school ADJ (belonging OR achievement OR success OR behav* OR performance* OR graduation OR learning OR engagement OR integration)) OR "high school" OR (educational ADJ (resilience OR attainment OR standard* OR status)) OR integration OR (learning ADJ (disorder* OR deficit OR difficult* OR disabilit* OR impairment OR problem* OR disturbance*)) OR "impaired learning").mp.)

Web of Science Core Collection
Science Citation Index Expanded, (SCI-EXPANDED)--1900-present Social Sciences Citation Index, (SSCI)--1900-present Arts & Humanities Citation Index, (AHCI)--1975-present Conference Proceedings Citation Index – Science, (CPCI-S)--1990-present Conference Proceedings Citation Index – Social Science & Humanities, (CPCI-SSH)--1990-present Book Citation Index – Science, (BKCI-S)--2005-present Book Citation Index – Social Sciences & Humanities, (BKCI-SSH)--2005-present Emerging Sources Citation Index, (ESCI)--2018-present Current Chemical Reactions, (CCR-EXPANDED)--1985-present Index Chemicus, (IC)--1993-present Search option: Exact search 318 results, September 09, 2022 TS=((unaccompanied AND (minor* OR child* OR adolescent* OR youth* OR young OR teen*)) AND (“peer relation*” OR socio-behav* OR sociobehav* OR (social NEAR/0 (behav* OR action OR activity OR activities OR contact* OR communication OR interaction* OR adapt* OR functioning OR responsiveness OR sensitivity OR health OR cognition OR exchange OR inclusion OR integration OR relation* OR competence* OR skill* OR abilit* OR disabilit* OR breakdown OR dysfunction OR handicap OR discrimination OR issues OR processes)) OR “adaptive behav*” OR (behav* NEAR/2 (disorder* OR disturbance* OR problem*)) OR externali* OR “conduct disorder*” OR ((“anti social” OR antisocial) NEAR/0 behav*) OR “socioemotional functioning” OR “human relation*” OR marginali?ation OR (interpersonal NEAR/0 (skill* OR competence* OR interaction* OR relation* OR communication)) OR adaptability OR flexibility OR maladjustment OR “social maladapt*” OR adjustment OR adaptation OR acculturation OR “cultural assimilation” OR “culture change” OR “sociocultural factor*” OR (“cross cultural” NEAR/0 (adapt* OR communication)) OR ((intercultural OR interethnic) NEAR/0 communication) OR “adaptive behav*” OR (academic NEAR/0 (achievement* OR underachievement OR success OR performance* OR failure*)) OR (school NEAR/0 (belonging OR achievement OR success OR behav* OR performance* OR graduation OR learning OR engagement OR integration)) OR “high school” OR (educational NEAR/0 (resilience OR attainment OR standard* OR status)) OR integration OR (learning NEAR/0 (disorder* OR deficit OR difficult* OR disabilit* OR impairment OR problem* OR disturbance*)) OR"

In constructing the research equation, we explored the population of UAMs in relation to their adaptative skills.

We included as key words, education and school performance, social abilities and behaviour problems. Resilience, acculturation and coping were also included as these terms are specific to the adaptative issues faced by UAMs in a foreign environment without the concrete and symbolic protection of adults.

### Study Inclusion Criteria

The inclusion criteria were peer-reviewed studies (A) published between 2005 and 2022; (B) at least partly observational and quantitative, with the exclusion of case reports (n < 10); (C) related to European data which permitted higher specificity (although we are aware that the asylum aspects and living conditions of refugees may vary even in this small geographical area); (D) published in English or French; (E) related to education, externalised disorders, social relationships and global adaptation of UAMs in European host countries. We decided to include qualitative literature reviews as our study aimed to draw an overview of the current knowledge on the adaptative skills and global functioning of UAMs. As milestones of previous research, reviews can indeed be of great help for a comprehensive understanding of earlier qualitative findings and trends.

### Population Inclusion Criteria

The studied populations had to be (A) children aged above 13 years; (B) described as unaccompanied refugees or migrants in the host country (studies relating to young adult refugees or migrants who arrived as UAMs in the host country but reached the age of majority during or just before the start of the study were also included. If no clear distinction was made between UAMs and AMs, the study was excluded); (C) forcibly displaced. We carefully checked the definition used by the studies that mentioned the term “migrant” as this can be very widely used. Thus, only papers referring to children not represented by an adult representative in the host country were included. As the term of unaccompanied minors is related to the situation of in the host country, we did not take into consideration the migration journey (alone or accompanied).

## Results

Our search strategy identified 1,050 articles. After eliminating duplicates, 778 articles were examined by title and abstract using the pre-defined inclusion and exclusion criteria. Deduplication of references exported from the databases was performed in Endnote 20 (Clarivate Analytics, United States).

**FIGURE 1:**
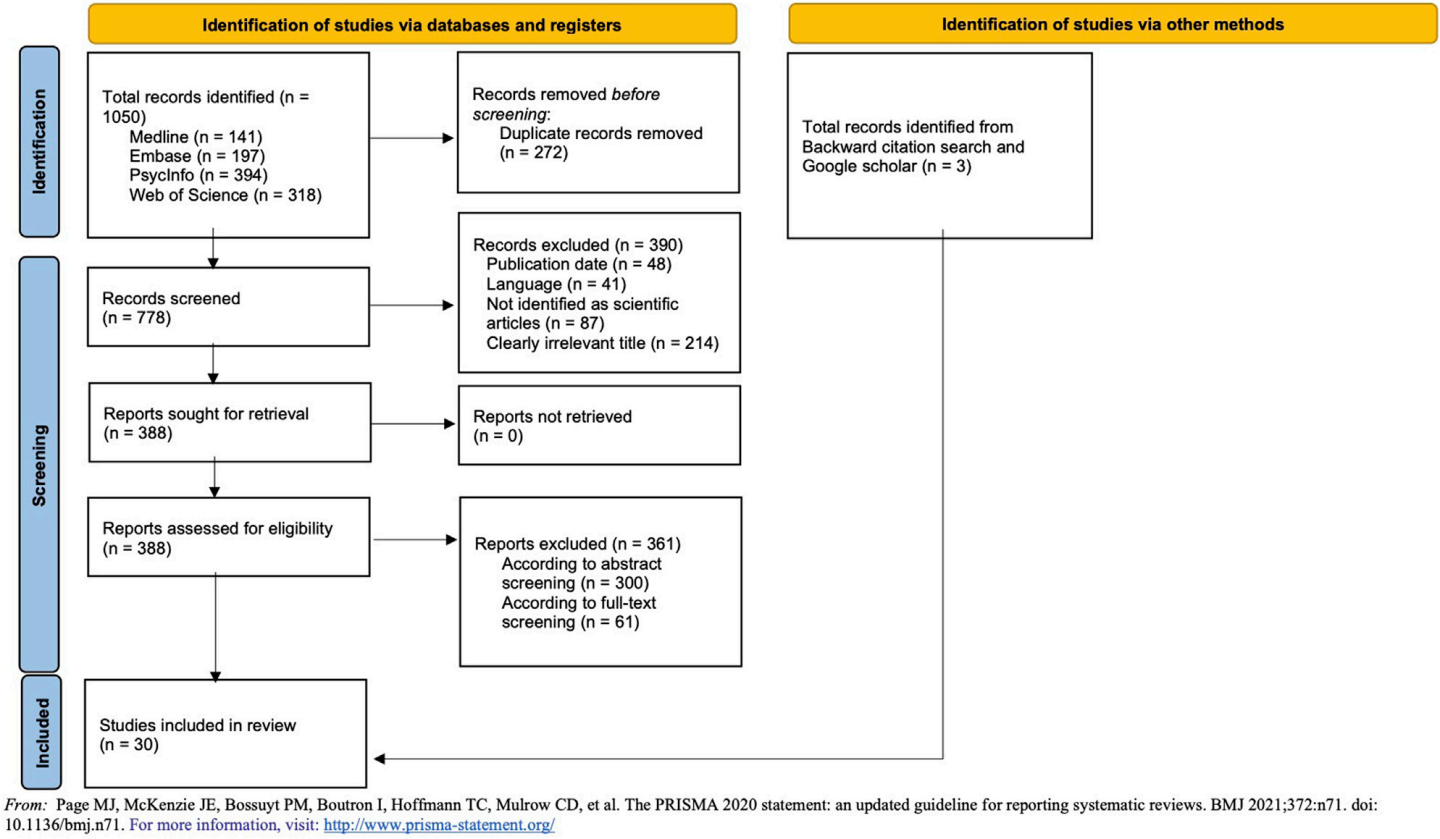
PRISMA 2020 flow diagram for new systematic reviews which included searches of databases, registers and other sources (Adaptative skills and global functioning of unaccompanied migrant minors in Europe: a systematised review, Lausanne, Switzerland, 2022–2024).

Twenty-seven articles were included using the search equation, and three additional studies were found by citation tracking and informal Google Scholar research. The PRISMA flow chart (Figure 1) summarises the selection process.

### Study Characteristics

Of the 30 studies, four were literature reviews, 12 used cross-sectional data with no control group, eight used cross-sectional data with a control group, four used longitudinal data with no control group, and two used retrospective data. Three of the studies were mixed (with qualitative and quantitative data).

Host countries in which research was conducted were as follows: Norway (n = 7), Belgium (n = 4), Austria (n = 4), the Netherlands (n = 3), Italy (n = 3), Spain (n = 2), Sweden (n = 1), Germany (n = 1) and the United Kingdom (n = 1). Literature reviews reported European and (partly) American data. Publication dates ranged from 2006 to 2022.

The size of the population examined ranged from 19 to 22,803 individuals. Control/comparison groups were mostly native-born youth (n = 5) or accompanied minors and immigrants (n = 3). One study compared two different groups of UAMs and one study compared UAMs to other children living in child care facilities.

In Norway, five of the reviewed studies were part of a single research program conducted by the Norwegian Institute of Public Health and included approximately 2,200 UAMs (or youth who arrived as such) and thus used highly similar data. The two other Norwegian studies used the same sample of UAMs. In the Netherlands, three studies were based on the same sample of 900 UAMs with different control groups (AMs, young migrants and social workers). In Austria, the three reviewed articles examined the same sample of 41 African UAMs between 15 and 18 years old.

In terms of measurement methods, self-report questionnaires for UAMs were mostly used (n = 24). Clinical data and structured interviews (n = 3) were also employed. A national register was used in the retrospective study.

Four studies collected data on the perceptions of interested parties (guardians, legal representatives, teachers) with regard to the UAM’s psychosocial health.

In terms of socio-demographic features, excluding the literature reviews, the overall number of UAMs in the reviewed studies reached approximately 25,000 UAMs. However, this was highly influenced by the Swedish retrospective study of 22,000 UAMs. Age was mainly between 15 and 18 years and male UAMs were over-represented in all studies. Globally, the most represented countries of origin were Afghanistan, Iraq (approximately 6700 UAMs put together), Somalia (approximately 2700 UAMs) and other African countries (nearly 2000 UAMs). Studies included in literature reviews had similar socio-demographic profiles.

The characteristics and results of the reviewed studies are detailed in Table 2.

**TABLE 2:** Table of results (Adaptative skills and global functioning of unaccompanied migrant minors in Europe: a systematised review, Lausanne, Switzerland, 2022–2024).

Authors, Study site Year of publication	Study type Population of interest	Outcomes Methods Focus	Findings	Limitations
	Literature reviews			
Wade, England, 2011 [27]	UAMs in England	• Response of social work services to UAM issues in Europe • Circumstances of preparation for adult life Narrative review: UAMs compared to other youth in social facilities Focus: Education and adaptation	• Old evidence (1995–2005): UAMs undergo high mobility and disruptions, which can negatively influence social relations and education • More recent studies (2007–2008) among social workers: effective engagement processes with UAMs which facilitated their resettlement • UAMs displayed high resilience and took advantage of personal and educational opportunities • The adaptation of UAMs tended to be better than that of local young people also in care when looking at placement, emotional/behavioural problems and educational achievement.	Unclear methodology
Ivert and Magnusson, Sweden, 2019 [28]	UAMs in Europe	• Drug use among UAMs • Criminality among UAMs Focus: Externalising disorders – substance use – social relations	• Substance abuse and criminality were not substantial problems among UAMs (maybe due to the lack of research) • Untreated mental health problems, stressful living conditions and lack of support and control can put UAMs at risk for substance abuse and criminality • Low levels of antisocial and externalising problems and high levels of internalising disorders (Oppedal and Idsoe, 2012; Derluyn and Broekaert, 2007) • Social workers reported more externalising disorders among UAMs than the UAMs themselves (Derluyn and Broekaert, 2007)	Some studies reviewed were published before 2004 (Manhica et al.) The group of interest was composed of minors within the social system Marginalised UAMs could be more likely to use substances and are not included in the group of interest.
Garcia and Birman, 2022, United States, Russia [29]	World literature with a predominance of European countries Qualitative and quantitative studies Body of literature covering the entire migration process, from the decision to migrate to settlement in the host country	• Decision to emigrate • Experiences of UAMs during the migration trip • Wellbeing and psychological distress at arrival • Effects of pre-migration traumas on post-migration distress • Aspirations • Adaptation, acculturation, social support Focus: Global adaptation – (education)	• Worsening of externalising disorders with time spent in the host country (Bronstein et al. 2013; Bean, Derluyn et al. 2007) • Acculturative stress and daily issues predicted depressive symptoms more significantly than war traumas before resettlement (Keles et al. 2016) • Qualitative data: High aspirations, willingness to look to the future rather than the past	High geographic heterogeneity
Aleghfeli and Hunt, United Kingdom, 2022 [30]	Systematic review: Europe and United States UAM in high-income countries	• Predictors of educational outcomes of UAM (risk and resilience factors) Focus: Education	• Risk and resilience factors were related to 5 socio-ecological levels: - Child - Microsystem - Mesosystem - Exosystem - Macrosystem • Micro and mesosystems played the most important role in educational resilience • Three sub-groups of UAMs were particularly disadvantaged: young mothers, minors who experienced detention during the immigration process and minors with pending or unknown immigration statuses	Ethnic factors were not studied Most studies were from the United States (which is not party to the 1951 Refugee Convention) In the included studies, it remained unclear whether the outcomes were the result of being a UAM or simply a refugee

Authors, Study site Year of publication	Study type Population of interest	Outcomes Methods Focus	Findings	Limitations
	Non-comparative cross-sectional studies			
Derluyn and Broekaert, Belgium, 2007 [11]	• 142 UAMs • Social workers and foster parents Comparison to literature data	Self-report questionnaires (*Hopkins Symptom Checklist-37 for Adolescents HSCL-37A, Strengths and Difficulties Questionnaire (SDQ)-self, Stressful Life Events Scale (SLE), Reactions of Adolescents to Traumatic Stress (RATS*)): • Socio-demographic features • Emotional and behavioural problems • Traumatic experiences Questionnaires for social workers or foster parents (*Child Behavior Checklist CBCL/6–18, SDQ-parent*): • Emotional or behavioural problems Focus: Internalising and externalising problems	• Higher levels of emotional problems among UAMs than among AMs (studied by Derluyn in 2005) • Self-reported levels of externalising problems were lower than those of AMs (Derluyn 2005) • Relatively good agreement between perceptions of UAMs and those of social workers regarding emotional and behavioural problems • Some severe internalising problems and peer problems were not detected by social workers	General limitation of cross-sectional studies: no causal relationship can be established General limitations of self-report questionnaires: Refugees may under-report deviant behaviour that could hinder their asylum process Varying cultural backgrounds in the study group: Differences in interpreting the questionnaires cannot be excluded
Bronstein et al., United Kingdom, 2013 [31]	222 Afghan male UAMs MNA	Socio-demographic features (Self-report questionnaires (*HSCL-37A, SLE*): • Emotional and behavioural problems • Stressful or traumatising life events Focus: Internalising and externalising problems	• 31.4% of UAMs scored in the high or very high scores for emotional and behavioural problems • 24.3% of UAMs had high or very high levels of internalising problems • Total number of stressful life events positively influenced externalising and internalising problems • Length of stay in the host country positively influenced scores of externalising problems	Cross-sectional studies (see limitations of Derluyn and Broekaert 2007) Self-report questionnaires (see limitations of Derluyn and Broekaert 2007) Only male UAMs The studied group came from only one London borough
Huemer et al., Austria, 2011 [32]	41 African UAMs (mostly males) Comparison to literature data	Self-report questionnaires and structured interviews • Psychiatric disorders (*Mini-International Neuropsychiatric Interview for Children and Adolescents*) • Emotional and behavioural symptoms (*Youth Self-Report*) • PTSD symptoms (*UCLA PTSD Reaction Index for DSM IV*) • Socio-demographic features (*Facts About You*) • Exposure to war Focus: Internalising and externalising problems – life and school satisfaction	• Levels of internalising problems and PTSD symptoms were higher among UAMs than among AMs and the general population • Very few externalising problems among UAMs • Behavioural and emotional functioning (*YSR*) were similar between UAMs of this study and general population data • UAMs were less satisfied in life than the general population, except for school	Cross-sectional studies (see limitations of Derluyn and Broekaert 2007) Self-report questionnaires (see limitations of Derluyn and Broekaert 2007) Small sample Varying home countries and reasons for migration Self-report questionnaires used were not validated in these cultural and language contexts
Huemer et al., Austria, 2013 [33]	Same sample as Huemer et al. (2011)	Self-report questionnaires: • Socio-emotional adjustment (*Weinberger Adjustment Inventory* (*WAI*)) • Emotional and behavioural symptoms (*YSR*) Focus: Internalising and externalising problems – Social relationships and aggressivity	• High levels of *repressive defensiveness, restrain* and denial of distress • UAMs reported high levels of distress and low levels of happiness	See Huemer et al. 2011
Völkl-Kernstock et al., Austria 2014 [34]	Same sample as Huemer et al. (2011)	Self-report questionnaires: • PTSD symptoms (*UCLA PTSD Reaction Index for DSM IV*) • Socio-demographic data, experiences during the flight, depression, anxiety and somatisation scale (*Scales for Children Affected by War and Persecution SAWP*) Focus: Social relationships and violence	• PTSD: see Huemer et al. (2011) • UAMs were shown to avoid interpersonal violence	See Huemer et al. 2011
Oppedal and Idsoe, Norway, 2012 [35]	556 UAMs (resettled between 2000 and 2009) Part of the *Youth, Culture and Competence Study (YCC)* of the Norwegian Institute of Public Health (NIPH)	Self-report questionnaires • Behavioural problems • Depression • Impact of war-related traumas • Acculturation problems • Cultural skills Focus: Internalising and externalising problems	• High levels of depression • Low levels of behavioural disorders • War-related traumas have a significant effect on depression symptoms but not on behavioural disorders • Time spent in the host country positively influences behavioural disorders	Cross-sectional studies (see limitations of Derluyn and Broekaert 2007) Self-report questionnaires (see limitations of Derluyn and Broekaert 2007) Varying cultural backgrounds in the study group: Differences in interpreting the questionnaires cannot be excluded
Jensen et al., Norway, 2015 [10]	93 UAMs (mostly males), mean length of stay at the time of study: 6 months	Self-report questionnaire • Stressful life events (*SLE measure*) • Psychologic symptoms (*HSCL*) • PTSD symptoms (*Child Posttraumatic Stress Disorder Symptom Scale CPSS*) Focus: Internalising and externalising problems	• Only a small number of UAM scored above the cut-off concerning externalising disorders • A number of SLEs were associated with PTSD, depression and anxiety symptoms but not externalising symptoms • Of those who answered the questionnaire about the impact of PTSD on daily functioning, one-third of UAMs reported no impact; 20% reported impact on five or more areas of functioning	Cross-sectional studies (see limitations of Derluyn and Broekaert 2007) Self-report questionnaires (see limitations of Derluyn and Broekaert 2007) Evaluation of externalising disorders (*HSCL*): question about alcohol or drug use were excluded from the *HSCL* questionnaire Only one-half of the participants answered the questionnaire about the impact of PTSD on daily functioning Different recruitment procedures depending on the host centre
Oppedal and Idsoe, Norway, 2015 [36]	896 former UAMs (mean length of stay: 3.5 years) Part of the *Youth, Culture and Competence Study (YCC)* of the Norwegian Institute of Public Health (NIPH)	Self-report questionnaires: • Depression (*CES-D for adolescents*) • *Impact of War-Related Traumatic Events (IWRTE)* • Social support • Cultural skills (*Host and Heritage Culture Competence Scale for Adolescents*) • Perceived discrimination • Socio-demographic features Focus: Social relationships – adaptation	• High levels of intrusion and depression symptoms related to war • UAM engaged in normative acculturation and socialisation processes	Cross-sectional studies (see limitations of Derluyn and Broekaert 2007) Self-report questionnaires (see limitations of Derluyn and Broekaert 2007) “The effects of culture competence may overlap with effects of other psychological constructs like IQ, cognitive and social skills”
Oppedal et al., Norway, 2017 [37]	Mixed study (qualitative and quantitative) Quantitative part: 918 former UAM (mostly males) Part of the *Youth, Culture and Competence Study (YCC)* of the Norwegian Institute of Public Health (NIPH) Same sample as Oppedal and Idsoe (2015)	Self-report questionnaires • Socio-demographic features • Educational aspirations • Depression symptoms (*Centre for Epidemiological Studies Depression Scale, CES-D for adolescents*) • PTSD symptoms (*Children’s Revised Impact of Event Scale-8, CRIES-8*) and trauma exposure • Perceived discrimination • Norwegian cultural competence Focus: Education and vocations	• No demographic variables or indicators of trauma, mental health and acculturation influenced educational aspirations • Despite high levels of mental health, traumas and adjustment problems, many UAMs engage in normal patterns of vocational development	Cross-sectional studies (see limitations of Derluyn and Broekaert 2007) Self-report questionnaires (see limitations of Derluyn and Broekaert 2007) *CES-D* et *CRIES-8* are not validated for clinics in Norway
Mueller-Bamouh et al., Germany, 2016 [8]	49 former male UAMs	Structured interviews: • Socio-demographic features • Exposure to family violence and organised violence (war and torture): *Checklist of Family Violence* and short version of *Vivo International Checklist of War, Detention and Torture events* • Traits of appetitive aggression and self-committed aggressive acts (*Appetitive Aggression Scale for Children (AAS-C)*) • PTSD symptoms (*UCLA PTSD Index for Children and Adolescents*) Focus: Social relationships and violence	• High prevalence of exposure to violence in the sample • Family violence and organised violence positively predicted the extent of PTSD symptoms • Family violence was positively associated with self-committed violent acts and appetitive aggression • Exposure to organised violence was not predictive of the occurrence of aggressive acts or appetitive aggression • Severity of PTSD symptoms had no influence on self-committed violent acts or appetitive aggressions	Cross-sectional studies (see limitations of Derluyn and Broekaert 2007) Selection bias: The sample could be a selection of individuals who have rejected violence and could be less likely to use violence than their peers who stayed in the home country
Longobardi et al., Italy, 2017 [38]	19 UAMs (18 men and one woman) who had experienced violence	Self-report questionnaires: • Behavioural problems, hyperactivity, emotional symptoms, peer problems, prosocial behaviour (*Strengths and Difficulties Questionnaire SDQ*) • PTSD symptoms (*Trauma Symptom Checklist for Children TSCC*) • Institutional violence (*ISPCAN Child Abuse Screening Tool Child Institution Version ICAST-CI*) • Resilience (*Child and Youth Resilience Measure CYRM-28*) Focus: Internalising and externalising problems – social relationships	• Scores of PTSD, depression and anxiety are above clinical cut-offs • No UAMs scored above the cut-off for behavioural problems • Scores of peer problems were in the borderline range • Scores of spiritual resilience were higher among UAMs	Cross-sectional studies (see limitations of Derluyn and Broekaert 2007) Self-report questionnaires (see limitations of Derluyn and Broekaert 2007) Very small sample Mostly males UAMs with severe psychiatric comorbidities were excluded from the study
Trenson et al., Belgium, 2022 [39]	27 UAMs in Belgian foster families Comparison to literature data	Self-report questionnaires: • *Reaction to Traumatic Stress* (*RATS*) • Emotional and behavioural problems (*SDQ*) • Resilience (*Resilience scale*) • *Transracial Adoption Parenting Scale (TAPS)* Focus: Internalising and externalising problems	• PTSD symptoms did not decrease with time spent in the host country • UAMs did not present more behavioural symptoms than their peers • UAMs who have contact with their parents had more behavioural problems	Cross-sectional studies (see limitations of Derluyn and Broekaert 2007) Self-report questionnaires (see limitations of Derluyn and Broekaert 2007) Small sample Questionnaires in Dutch: Comprehension problems

Authors, Study site Year of publication	Study type Population of interest	Outcomes Methods Focus	Findings	Limitations
	Comparative cross-sectional studies			
Bean et al., Netherlands, 2006 [40]	Cross-sectional data part of a larger comparative longitudinal study • 920 UAMs • Control group: 1,059 native Dutch adolescents Guardians and teachers included	Self-report questionnaires among UAMs, guardians and teachers: • Internalising and externalising disorders (*HSCL-37A*) • Stressful life events (*SLE checklist*) • PTSD reactions as defined in DSM-IV (*RATS*) • *Mental Health Questionnaire for Adolescents* • *Mental Health Questionnaire for Guardians* • *Mental Health Questionnaire for Teachers* • Emotional and behavioural problems of UAMs as perceived by their guardians: *Child Behavior Checklist* (*CBCL*) • Emotional and behavioural problems of UAMs as perceived by their teachers: *Teacher’s Report Form* (*TRF*) Focus: Internalising and externalising problems (self-reported and perceived)	• UAMs had the highest scores of internalising disorders, symptoms of PTSD and SLE • Native adolescents had the highest scores of externalising disorders • Guardians and teachers detected emotional distress and mental healthcare needs only in a small proportion of UAMs (21% and 30%, respectively) • The use of mental care services was mostly determined by the perceptions of guardians and teachers but not by the needs reported by UAMs • 48.7% of UAMs reported unmet mental health needs (vs. 4.5% of Dutch adolescents)	Cross-sectional studies (see limitations of Derluyn and Broekaert 2007) Varying cultural backgrounds in the study group: Differences in interpreting the questionnaires cannot be excluded
Bean et al., Netherlands, 2007 [9]	• 1,110 UAMs (920 of whom were already in Bean et al., 2006) • New: 1,187 non-Dutch speaking immigrants in Belgium • 917 native Dutch adolescents	Self-report questionnaires: • Internalising and externalising disorders (*HSCL-37A*) • *SLE checklist* • PTSD reactions as defined in DSM-IV (*RATS*) Focus: Internalising and externalising problems	See Bean et al. 2006	Cross-sectional studies (see limitations of Derluyn and Broekaert 2007) Self-report questionnaires (see limitations of Derluyn and Broekaert 2007) The study presents the same results as the 2006 study regarding the prevalence of internalising and externalising disorders. New features are also developed but are not relevant to our research question
Derluyn et al., Belgium, 2008 [5]	• 1,219 adolescent non-Dutch speaking immigrants (same as Bean et al. 2007): 103 are UAMs • 607 Belgian adolescents	Self-report questionnaires: • Internalising and externalising disorders (*HSCL-37A*) • Behavioural problems, hyperactivity, emotional symptoms, peer problems, prosocial behaviour (*Strengths and Difficulties Questionnaire SDQ*) • *SLE checklist* • PTSD reactions as defined in DSM-IV (*RATS*) Focus: Internalising and externalising problems	• UAMs had lower scores of conduct disorders than AMs • UAMs scored higher regarding emotional disorders and PTSD	Cross-sectional studies (see limitations of Derluyn and Broekaert 2007) Self-report questionnaires (see limitations of Derluyn and Broekaert 2007) Population and findings are probably highly similar to those of Bean et al. 2006–2007
Thommessen et al., Italy, 2013 [41]	Parents/caregivers or social workers of 60 male UAMs (mean length of stay: 2 years) and 60 Italian male adolescents	Questionnaire completed by legal representatives or referring social workers: • *Child Behavior Checklist* (CBCL) Focus: Perceived internalising and externalising problems	UAMs were more likely to be classified with internalising and externalising problems than their Italian peers	Cross-sectional studies (see limitations of Derluyn and Broekaert 2007) Perceived mental health was not compared to self-report data
Natalucci et al., Italy, 2020 [7]	• 98 male UAMs, half of them from Egypt • 103 Italian adolescents	Self-report questionnaires • *Strengths and Difficulties Questionnaire* (*SDQ*) Focus: Internalising and externalising problems	• UAMs scored significantly higher in emotional and peer problems compared to Italian adolescents • Non-Egyptian UAMs scored lower in emotional and peer-related problems than Egyptian UAMs • Italian adolescents scored higher in behavioural problems than UAMs • No UAMs scored in the abnormal range of prosocial behaviour (vs. 9.7% of Italian adolescents) • No differences between both groups on the hyperactivity scale • No differences between both groups regarding total difficulties	Cross-sectional studies (see limitations of Derluyn and Broekaert 2007) Self-report questionnaires (see limitations of Derluyn and Broekaert 2007) Small sample Half of the MNA sample are Egyptian minors: this percentage does not represent the reality of the Italian asylum context
González-García et al., Spain, 2017 [42]	1,216 children living in residential child care: 93 UAMs The authors distinguished two groups with specific needs: UAMs and children with intellectual disabilities. These two groups are compared to the general sample	Self-report questionnaires and clinical features: • Socio-demographic and familial features • Mental health features (*CBCL* and by questioning therapists) • School functioning Focus: Externalising disorders - education	• Clinically, there were lower levels of mental difficulties among UAMs • Only one-third of the unaccompanied immigrant minors were in compulsory education (vs. 80% of the general sample) • UAMs scored higher regarding school motivation and lower regarding behavioural problems	Cross-sectional studies (see limitations of Derluyn and Broekaert 2007) UAMs were compared to a group of children in residential care and not to the general population
Martinez-Martinez et al., Spain, 2021 [43]	292 UAMs Comparison between UAMs resettled for more versus less than 9 months	Self-report questionnaires: • Socio-demographic features • *Personal Learning Environment* (PLE) • *Self-Efficacy* Focus: Education – long-term functioning	• UAMs resettled for more than 9 months had better academic planning and management as well as improved use of educational resources • Communication and social interaction variables were lower in the group resettled for more than 9 months	Cross-sectional studies (see limitations of Derluyn and Broekaert 2007) Self-report questionnaires (see limitations of Derluyn and Broekaert 2007) No comparison to the general population
Seaman and Stites, Austria, 2022 [44]	Mixed study 72 former UAMs and AMs from Iraq, Syria and Afghanistan resettled between 2014 and 2019: 37 UAM	Self-report questionnaires (quantitative): employment, education, socio-demographic features Individual interviews (qualitative) for 31 youths Focus: Education – social relationships – long-term functioning	• UAMs were shown to secure employment faster, establish more diverse social connections and acquire German language skills faster than AMs • A higher percentage of UAMs enrolled in education than AMs (73% vs. 43%) • AMs were more likely to pursue their education in university (67% vs. 19%) • The need for financial autonomy pushed UAMs to choose paid training paths (apprenticeships rather than university). This decision is a significant post-migration stress factor • UAMs had a greater sense of belonging in Austria Qualitative data: UAMs had high career ambitions	Cross-sectional studies (see limitations of Derluyn and Broekaert 2007) Self-report questionnaires (see limitations of Derluyn and Broekaert 2007) Self-report questionnaires used were not specified

Authors, Study site Year of publication	Study type Population of interest	Outcomes Methods Focus	Findings	Limitations
	Retrospective studies			
Vervliet et al., Belgium, 2015 [45]	Mixed study (retrospective and cross-sectional; quantitative and qualitative) 52 Afghan UAMs newly resettled in Belgium	Self-report questionnaires: • Personal and familial aspirations during the pre-migration period (examined retrospectively): *Aspiration Scale for Refugees and Migrants* (*ASRM*) • Same procedure regarding aspirations during the post-migration period (quickly after arrival) Semi-structured interviews (qualitative) Focus: Education	• Educational and professional aspirations were the second most important pre-migration aspirations after the search for a safe environment • The motivation to find a safe environment was closely linked with educational aspirations • In the post-migration period, educational and administrative aspirations (obtaining a residence permit) came first	Cross-sectional studies (see limitations of Derluyn and Broekaert 2007) Self-report questionnaires (see limitations of Derluyn and Broekaert 2007) Retrospective data collection on pre-migration aspirations is likely to be influenced by experiences during the journey Participants were exclusively Afghan: the political context of the country influences the aspirations in an important way
Çelikaksoy and Wadensjö, Sweden, 2019 [46]	• Every young adult refugee (19–27 y.o.) who resettled as UAMs between 2003 and 2014 in Sweden (n = 22,000) • AM youths resettled in the same period (19–27 y.o.) • Young natives (19–27 y.o.)	Wellbeing in the labour market: • Employment: yes/no • Insecure employment Focus: Education – Employment	UAMs were more likely to complete their education at a later age and to use alternative educational pathways (e.g., adult education)	No data on hourly wages: The results regarding precarious jobs could be because UAMs work more hours or have several jobs. This would call into question the results on labour market wellbeing.

Authors, Study site Year of publication	Study type Population of interest	Outcomes Methods Focus	Findings	Limitations
	Non-comparative longitudinal studies			
Bean et al., Netherlands, 2007 [47]	• 920 UAMs at T1, • 582 UAMs at T2 • Teachers and guardians included Same sample as Bean et al. (2006, 2007) cross-sectional studies About 12 months between T1 and T2	Self-report questionnaires • Internalising and externalising problems (*HSCL-37A*) • *SLE checklist* • PTSD reactions as defined in DSM-IV (*RATS*) Questionnaires for guardians and teachers: • *Child Behavioural Checklist Guardian report CBCL* • *Teacher’s Report Form* Focus: Internalising and externalising problems (self-reported and perceived)	• Total number of SLE-predicted PTSD reactions, emotional distress and behavioural disorders in self-report questionnaires • Low level of concordance between self-reported and perceived problems	Data collection at T1 was in 2002 Varying cultural backgrounds in the study group: Differences in interpreting the questionnaires cannot be excluded It cannot be excluded that highly traumatised individuals were under-represented in the group taking part in the study
Keles et al., Norway, 2017 [48]	- T1 : 918 UAM (>13 y.o.) - T2 : 580 UAM - T3 : 229 UAM Mean length of 1.41 years and 1.26 years between data collection Part of the *Youth, Culture and Competence Study (YCC)* of the Norwegian Institute of Public Health (NIPH)	Self-report questionnaires at T1, T2 and T3 • Depressive symptoms (*CES-D*) • General hassles: finances, conflicts, school achievement, relationships • Acculturation hassles • Impact of war-related traumas • Demographic features Focus: Adaptation and global functioning	• Acculturation hassles predicted depressive symptoms, but depressive symptoms did not predict acculturation hassles • There was a bidirectional relationship between depressive symptoms and general hassles • The level of depression did not change over time, but the levels of general difficulties and acculturation decreased	Self-report questionnaires (see limitations of Derluyn and Broekaert 2007) Over-representation of male and Afghan participants compared to the overall group of UAMs Difficulty to reach the sample at T2 and T3 (high mobility, change of phone number, loss of sight, *etc.*)
Jensen et al., Norway, 2019 [49]	- T1 (6 months after arrival): 95 UAMs – same sample as Jensen et al. (2015) - T2 (2 years after arrival): 78 (former) UAMs - T3 (5 years after arrival): 47 (former) UAMs	Self-report questionnaires: • Internalising and externalising problems (*HSCL-37A*) • PTSD symptoms (*CPSS*) • Somatisation (*Children’s Somatisation Inventory Short Form CSSI-8*) • *SLE checklist* • *Daily Stressors Scale for Young Refugees* (*DSSYR*) • Social support social (*Duke-UNC Functional Social Support Questionnaire FSSQ*) Focus: Internalising and externalising problems	• Female and highly traumatised UAMs had higher levels of depression, anxiety and PTSD symptoms. This was not true for externalising disorders • At T3, levels of depression had decreased but not levels of PTSD, anxiety and externalising disorders • Daily stressors had a significant influence on mental health problems	Self-report questionnaires (see limitations of Derluyn and Broekaert 2007) Only half of the participants were still in the study at T3 High degree of individual variability between times of data collection
Sleijpen et al., Norway, 2022 [50]	See Keles et al. 2017	Self-report questionnaires • Attachment patterns*: Experiences in Close Relationships – Relationships Structure questionnaire (ECR-RS)* • Presence of adult confidants at T1 • Socio-demographic features Focus: Social relationships – long-term functioning	• Avoidance patterns increased over time spent in Norway • Male UAMs were more likely to express avoidant attachment patterns • Initial levels of avoidance patterns were lower among UAMs who reported having at least one adult confidant • Levels of anxiety did not decrease over time spent in Norway	See Keles et al. 2017

### Education and School Performance

Regarding education, we took in consideration studies that reported data about school success as well as educational pathways and environment of UAMs. Grades, motivation and self-management at school, achieving higher education (university) and safe employment as well as aspirations are examples of variables that were reported in the reviewed studies to describe experiences of UAMs in educational settings.

In total, UAMs’ educational environment and performance were addressed in 13 of the included records. Several authors found that UAMs were disadvantaged in terms of attaining higher education and safe employment [27, 42, 46]. In spite of this trend, a high capacity for resilience was also described in the same studies, with UAMs showing similar performances to other youth in social care [27] or using alternative paths to achieve their educational and professional goals [42, 46].

According to some authors, UAMs expressed their resilience (defined in 2 studies as “competence under stress” and “utilizing resources and behaviours to overcome challenges and maintain and achieve wellbeing” [51, 52]) through high levels of motivation and aspirations [29, 44], regardless of their trauma experiences and levels of psychopathology [37, 42].

One study showed education and employment to be the second-highest pre-migration and the highest post-migration aspiration in Afghan UAMs [45]. Another study showed that UAMs’ school satisfaction was better than that of the general population [32].

Protective factors for the academic achievement of UAMs were analysed in one systematic literature review [30]. The authors separated resilience from risk factors according to five “socio-ecological” levels: the child (individual characteristics), micro and meso-systems (socio-administrative, individual and community support), exosystems (employment and finances) and macrosystems (migration policy, community). Globally, micro and mesosystemic influences (i.e., supportive or unsupportive parents and teachers, supportive friends or disruptive classmates) were shown to be the most important resilience factors among the UAM population. Furthermore, the European evidence drew particular attention to specific disadvantaged sub-groups of UAMs: UAMs with mental or physical health issues [30, 53], young UAM mothers, UAMs who have lived in detention and UAMs with unknown or pending asylum status [30].

Two studies highlighted the ability of UAMs to successfully use efficient educational resources quickly after arrival [43, 44].

One Austrian study found that because of their need for self-sufficiency, UAMs were less likely to be involved in university education than their AM peers [44].

Finally, concerning the school environment, two studies found that UAMs’ mental health needs were often underestimated and under-reported by teachers (same authors and same population) [40, 47].

### Social and Interpersonal Skills

We used the APA definition for interpersonal skill which is an “aptitude enabling a person to carry on effective interactions and relationships with others, such as the ability to communicate thought and feeling or to assume appropriate social responsibilities” [6]. In our review, we also included studies reporting data on aggressive behaviours, antisocial functioning or conduct disorders as they are part of interacting with others.

Nine studies addressed the social and interpersonal skills of UAMs, as well as the prevalence of violent or aggressive behaviours. One study addressed the social functioning of 41 African UAMs resettled in Austria [33]. High levels of *repressive defensiveness* (“extreme self-restraint or suppression of egoistic desires”) and *restraint* (“impulse control, suppression of aggression, consideration of others and responsibility”) were observed. In another study based on the same sample of UAMs, a low level of interpersonal violence was found [34]. In a German study of 49 men who were former UAMs, family violence (in the premigration period) predicted more aggressive actions, whereas exposure to organised violence (war, torture) had no impact on the occurrence of such acts nor on appetitive aggression (a propensity to “violence-related enjoyment”) [8]. Furthermore, the severity of posttraumatic stress symptoms did not influence aggressivity and the number of violent acts committed [8].

Peer relationships were assessed in two Italian studies [7, 38]. Scores of peer-related problems were found to be at increased levels or in the borderline range (compared to clinical scores and native youth). Despite this, studies addressing prosocial behaviour and socialisation (through self-reported questionnaires) showed that UAMs usually scored in the normal range of these variables, and sometimes better than AM youth [7, 36, 44].

Long term outcomes in communication and social skills of UAMs living in Spain for 9 months were reported as worse than those of UAMs who resettled more recently [43]. This is in line with Norwegian data showing more avoidance in UAMs as time goes by since resettlement [50]. The authors of this last study noted higher avoidance behaviours among male UAMs and lower avoidance behaviours among UAMs who reported being in touch with an adult confidant [50].

### Externalising Disorders

In opposition to internalising disorders defined as a psychiatric condition involving “overinhibited or internally focused symptoms” such as anxiety disorders, fear, and depression, externalising disorders refer to a spectrum of disinhibition and external behaviours such as aggressivity, delinquency, conduct problems, oppositionality, hyperactivity and attention problems [54].

From an epidemiological perspective, it is worth questioning the prevalence of externalising disorders as UAMs have been shown to internalise mental health issues. Seventeen reviewed studies covered the prevalence, expression and evolution of externalising disorders in UAMs. Externalisation and behavioural problems have frequently been compared to internalisation and emotional disorders. As mentioned in the introduction, UAMs’ “external functioning” is less likely to be impaired than their “internal functioning” [5, 10, 32, 33, 38].

Reviewed epidemiological studies comparing the prevalence of externalising disorders in UAMs vs. AMs and native youth are listed in Table 3.

**TABLE 3:** Externalising disorders, epidemiological studies (Adaptative skills and global functioning of unaccompanied migrant minors in Europe: a systematised review, Lausanne, Switzerland, 2022–2024).

	Higher prevalence among UAMs	Same or non-upper prevalence	Lower prevalence among UAMs
Externalising disorders: **UAMs vs. AMs**	*Thommessen et al.* 2013 (hetero-reported data) [41]	*Derluyn and Broekaert.* 2007 [11]	*Derluyn et al.* 2008 [5]
Externalising disorders: **UAMs vs. native youth**		• *Huemer et al.* 2011–13 [32, 33] • *Trenson et al.* 2022 [39]	• *Derluyn et al.* 2008 [5] • *Bean et al.* 2006–2007 [40, 47] • *Natalucci et al.* 2020 [7] • *González-García et al.* 2017 [42]

The overall result is a lower, although sometimes identical, prevalence of externalising disorders in UAMs compared to control groups. In an Italian study of a small sample of UAMs who had experienced violence, behavioural problems as measured by scores on the *Strength and Difficulties Questionnaire* remained below normative clinical scores [38]. It is noteworthy that the only study that showed a higher prevalence of externalising disorders among UAMs was based only on problems perceived by caregivers and social workers [41].

The findings of the reviewed studies (which show high methodological similarities) are heterogenous concerning the link between past trauma and externalising disorders. Past trauma did not influence the prevalence of these disorders or the socialisation and acculturation processes in four studies [10, 35, 36, 49] while they did in tree others [31, 47].

Surprisingly, two studies highlighted an increase in the prevalence of behavioural disorders which are part of the externalisation spectrum, with time spent by UAMs in the host country [31, 35]. A more recent work showed a similar increase in behavioural disorders in UAMs especially when they had contact with their parents [39].

Some authors reviewed the European literature with an interest in substance abuse and criminality among UAMs. They reported the same trends as in our work concerning the prevalence of externalising disorders among UAMs and AMs [28]. The authors failed to show a clear picture concerning substance use among UAMs but highlighted that post-migration risk factors were likely to increase drug consumption and criminality in this population [28].

### Global Functioning and Long Term Adaptation

Several studies included in our review addressed the short and long term functionality of UAMs in view of the high prevalence of mental health conditions/disorders (or the high level of psychopathology). Global functioning is defined by the World Health Organisation (WHO) as “the positive features of the relationship between a health condition and the environmental and personal context of the individual” [55]. It can thus be related to functioning in different areas of life and can be measured with different scales.

Despite some contrasting results, our review showed that the overall good functioning of UAMs in educational and social spheres is likely to apply to other areas. In a Norwegian cross-sectional UAM study, one-third of those with PTSD were not impacted in their functioning, while 20% reported significantly impaired functioning [10].

Furthermore, despite high levels of depressive disorders, longitudinal study showed no influence of depression on UAMs’ acculturation (assimilation of culture in the host country) [48]. However, several of the reviewed articles showed that in the long-term disorders present on arrival were likely to become chronic [29, 39, 48–50]. In some studies, externalising disorders that were initially absent were likely to develop with more time spent in the host country [28, 31, 50].

## Discussion

This systematised review contributes to the current state of knowledge on adaptative skills and global functioning of UAMs. It appeared that in the European context, the adaptative capacities of UAMs are largely preserved despite a high prevalence of PTSD, depression and anxiety. These results illustrate a type of resilience with preserved functional capacities and strong motivation to succeed in academic and social life. As pointed out by several authors, UAMs suffer from largely internalised disorders, which creates a gap between the emotional distress they experience in their private lives and the resilient behaviours they express in the social spheres.

The few longitudinal studies involving UAMs make this observation even more complex by showing a gradual decline in adaptative behaviour related to the length of time spent in the host country. This issue should be addressed in further longitudinal studies.

### Scholarly and Social Resilience Among UAMs

It was clear in our review that UAMs show particular resilience in facing the challenges of migration and the settlement process. This has been the subject of many European qualitative studies, which are not included in our review [56–58]. Different contextual and societal factors may explain these results. First, it seems obvious that UAMs are likely to undergo particular pressure to integrate and excel in educational, professional and social domains. Successful integration can lead to better social inclusion in the host country [27, 59], the ability to support oneself [42, 58] and, in some cases, to provide for the needs of the family in the home country [57]. While facing chaotic life circumstances resulting from migration, insecurity in the asylum process and a future that may seem doomed, UAMs might consider academic and social achievement as a way to control their life and an important resilience strategy [60, 61].

This great maturity of UAMs in scholarly and social spheres of functioning is described by one study as part of an “adultomorphic behaviour” they adopt in response to the absence of parents [7]. During and after migration, these young people may be forced to adopt such behaviour to provide for their needs without any adult protection. Furthermore, as a way to take up the space left by the absence of parents UAMs may engage in the creation of more social connections and integrate quicker than their AM peers [44].

Nevertheless, as some authors mentioned, a selection effect in the great resilience of the UAM population in Europe is highly probable. Those who make it to Europe may represent a particularly resilient population, explaining their strong ability to cope with the difficulties of post-migration life [32]. This same effect could also be observed concerning violent behaviours in youth who have experienced organised violence [8]. According to these authors, the studied sample of refugees could be a selection of individuals who rejected war, with those who stayed behind being more likely to have adapted to the violent environment and consequently developed violent behaviours [8].

### Externalising Disorders in the Migration Context

Studies conducted in host countries mostly showed preserved external functioning and an equal or lower level of social and behavioural disorders than in AMs [11] and natives [5, 7, 9, 32, 33, 40].

Reviewed articles suggest low association between UAMs’ psychopathologies and school motivation, aspirations and social skills [37, 42]. Nevertheless, this finding must be carefully interpreted as it could lead to underestimating the real mental health needs of this population. This tendency was especially noted in studies comparing self-reported and perceived (by an adult in charge) mental health needs of UAMs [40, 47]. Indeed, high levels of unmet needs were alarming and may contribute to the chronification of disorders already present from the moment of arrival [47–50].

Although we might expect time spent in the host country to have a positive effect, the few longitudinal studies reported here surprisingly showed higher levels of chronic disorders that altered previously acquired good social functioning. Socio-behavioural problems, even when they were absent at the time of arrival, appeared or worsened with the length of stay in the host country [31]. Peer influence, in that context, could either be a protective or a risk factor [31, 39] and should be addressed in further studies.

However, it is reasonable to assume that the approach of age of adulthood may explain new behavioural problems as it implies a greater risk for UAMs to be sent back. They may be exposed to a feeling of loss of control [31], and present reactive behaviours in that context. From another point of view, this situation of insecurity could also lead UAMs to conceal their disorders to meet the social and administrative requirements imposed by the asylum process [32].

Good global functioning, which was described in the educational, professional and social spheres, as well as the initial low level of externalising disorders, should then be carefully considered. The legal and administrative contexts in which UAMs find themselves are likely to be a source of stress and anxiety, maintaining emotional difficulties at a high and stable level or causing more externalising disorders [7, 28, 31, 33, 35, 62].

Thus, our review draws particular attention to the post-migration risk factors that UAMs face while resettling in a new country. By only focusing on their pre- or peri-migration background, mental health professionals may miss a whole sphere of influence on the overall experience of UAMs [28].

### Adaption and Resilience: Post-migration Period and Paradoxes

Our review contributes to the field of knowledge of adaptative and global functioning of UAMs in Europe. We showed that UAMs displayed a specific form of resilience in terms of academic, social and externalising behaviours. These results have been discussed in the previous chapters but it is worth considering them in the light of the status of UAMs in host countries. In Western societies, this status is at the border of several concepts. On the one hand, an UAM is considered as a child whose “best interests,” should be protected (as defined by the United Nations Convention on the Rights of the Child); on the other, as an asylum seeker subject to the relevant decision-making processes. Public institutions are likely to exert indirect pressure on UAMs by requiring them to adapt quickly and effectively, as they risk being sent back on reaching adulthood. This paradox between reception and integration pressure, as well as between child protection and asylum procedures, was raised by several authors and experts in this domain [62–64] and may explain our results.

The high marginalisation of UAMs should be set in the context of the insufficient support they receive in the host country [62] and the good level of functioning they must adopt by hiding their disorders and meeting the challenges of integration. In one review, it was demonstrated that the same study can show a UAM to be “vulnerable” [65], resilient [66] or both at the same time [29]. This strengthens the tension between precariousness and perseverance which is specific to this group.

In sum, the lack of knowledge or denial of these paradoxical issues by public institutions, represents a significant danger to the wellbeing of UAMs on reaching adulthood.

### Limitations

First, because Europe is a large geographical area, it is not possible to generalise any migration policy, resettlement context or access to care for UAMs. The conclusions of this review should thereby be considered carefully. Nevertheless, the studies reviewed focused on the Western and Northern European countries which are host to a large majority of the UAMs in Europe.

Studies of UAMs display high heterogeneity in the populations selected, selection procedures (most samples are unrepresentative convenience samples), sample sizes and diagnostic procedures (interview, various self and hetero-reported scales).

UAMs typically come from different countries and cultures, which may have influenced the findings, especially when self-report questionnaires were used. UAMs from different cultural backgrounds are likely to differ in their perceptions of difficulties and the socio-administrative context they are facing. Furthermore, the diversity of languages and causes of migration are other factors that may influenced the results of this review. Therefore, it would be too simplistic to consider that the journey of UAMs in our Western societies can be perfectly predicted and categorised.

According to some authors, self-report questionnaires may introduce potential selection bias by underestimating externalising disorders. This is what sometimes called the *social desirability bias* [30], which can be amplified in the context of administrative insecurity, such as asylum status, and can lead UAMs to hide externalising disorders [32, 63]. Furthermore, regarding the term “externalising disorders,” it should be noted that the selected studies seldom analysed the subtypes of this broad category within the UAM population.

Finally, this review highlighted the lack of longitudinal studies, which provide the most reliable information on the social and mental health outcomes of UAMs.

### Conclusion

This study aimed to conduct a systematised review of adaptative skills and global functioning of UAMs, a population who is subjected to migration, family separation and mental health issues.

Our result showed that UAMs have great motivation at school and high professional aspirations. When presenting mental health disorders, they tend to conceal their dissocial and behavioural manifestations. This is reflected in high scores of socialisation, low levels of externalising disorders and only little or no use of violence.

The high prevalence of anxiety, depression and posttraumatic stress disorders reflects a process of internalisation of mental health issues and can explain this population’s “external life” success. Despite the strong evidence concerning this form of external resilience that leads to a good global adaptation of UAMs, domains of life satisfaction and personal fulfilment remain to be explored. As pointed out in one of our study, resilience is a multidimensional concept that may be independent of mental health issues [16].

Finally, it should be noted that stress and difficulties experienced after settling in the host country may negatively influence mental health in the same way (and sometimes to a greater extent) as pre- and peri-migration risk factors. Post-migration stressors can lead to a chronification of disorders. Findings from longitudinal studies should raise awareness of the development and persistence of mental disorders in UAMs long after their arrival. When meeting these minors in care facilities, particular attention should be paid to dealing with ongoing or new psychosocial stressors and not only to the traumatic elements experienced before arrival [67, 68].
